# Dose Tailoring of Vancomycin Through Population Pharmacokinetic Modeling Among Surgical Patients in Pakistan

**DOI:** 10.3389/fphar.2021.721819

**Published:** 2021-11-11

**Authors:** Muhammad Muaaz Munir, Huma Rasheed, Muhammad Imran Khokhar, Rizwan Rasul Khan, Hafiz Asad Saeed, Mateen Abbas, Mohsin Ali, Rabiea Bilal, Hafiz Awais Nawaz, Abdul Muqeet Khan, Shaista Qamar, Syed Muneeb Anjum, Muhammad Usman

**Affiliations:** ^1^ Institute of Pharmaceutical Sciences, University of Veterinary and Animal Sciences, Lahore, Pakistan; ^2^ Ameer-ud-Din Medical College, Post-Graduate Medical Institute (PGMI), Lahore General Hospital, Lahore, Pakistan; ^3^ Department of Medicine, Aziz Fatima Medical and Dental College, Faisalabad, Pakistan; ^4^ Pak Emirates Military Hospital, Rawalpindi, Pakistan; ^5^ Quality Operation Laboratory, University of Veterinary and Animal Sciences, Lahore, Pakistan; ^6^ Department of Pharmacy Practice, Faculty of Pharmaceutical Sciences, Govt College University, Faisalabad, Pakistan; ^7^ CMH Lahore Medical College and IOD, NUMS, Lahore, Pakistan

**Keywords:** vancomycin, population pharmacokinetics, Pakistan, NONMEM, dose tailoring

## Abstract

**Background:** Vancomycin is a narrow therapeutic agent, and it is necessary to optimize the dose to achieve safe therapeutic outcomes. The purpose of this study was to identify the significant covariates for vancomycin clearance and to optimize the dose among surgical patients in Pakistan.

**Methods:** Plasma concentration data of 176 samples collected from 58 surgical patients treated with vancomycin were used in this study. A population pharmacokinetic model was developed on NONMEM^®^ using plasma concentration–time data. The effect of all available covariates was evaluated on the pharmacokinetic parameters of vancomycin by stepwise covariate modeling. The final model was evaluated using bootstrap, goodness-of-fit plots, and visual predictive checks.

**Results:** The pharmacokinetics of vancomycin followed a one-compartment model with first-order elimination. The vancomycin clearance (CL) and volume of distribution (Vd) were 2.45 L/h and 22.6 l, respectively. Vancomycin CL was influenced by creatinine clearance (CRCL) and body weight of the patients; however, no covariate was significant for its effect on the volume of distribution. Dose tailoring was performed by simulating dosage regimens at a steady state based on the CRCL of the patients. The tailored doses were 400, 600, 800, and 1,000 mg for patients with a CRCL of 20, 60, 100, and 140 ml/min, respectively.

**Conclusion:** Vancomycin CL is influenced by CRCL and body weight of the patient. This model can be helpful for the dose tailoring of vancomycin based on renal status in Pakistani patients.

## Introduction

Vancomycin has been used for more than six decades and is predominantly effective for the treatment of infections caused by methicillin-resistant *Staphylococcus aureus* (MRSA), methicillin-susceptible *Staphylococcus aureus* (MSSA), and *Staphylococcus epidermidis* (Zhao et al., 2014). Plasma levels of vancomycin below the target trough concentration may cause insufficient eradication of bacteria, while overdosing may lead to toxicity (Ingram et al., 2008). The therapeutic trough concentration of vancomycin is a crucial pharmacokinetic metric to achieve the antibacterial effect and reduction in microbial resistance. Therefore, therapeutic drug monitoring (TDM) is recommended for the safe and effective outcomes of vancomycin treatment (Martin et al., 2010). The recently published consensus guidelines by the American Society of Health-System Pharmacists (ASHP), Infectious Diseases Society of America (IDSA), the Pediatric Infectious disease Society (PIDS), and the Society of Infectious Diseases Pharmacists (SIDP) have suggested vancomycin trough concentrations of 15–20 mg/l for most infections (Rybak et al., 2020).

Age-related differences in the pharmacokinetics of vancomycin in pediatric and adult patients necessitate dose adjustment. Developmental changes in kidney function also affect the serum concentrations of drugs that are renally excreted, as in the case of vancomycin, where 80%–90% of the drug is excreted *via* renal elimination (Matzke et al., 1984; Rodvold et al., 1988; Ducharme et al., 1994). Therefore, the kidney status of patients needs special attention for proper vancomycin dosing, particularly in elderly patients exhibiting a progressive decline in kidney function with increasing age (Verhave et al., 2005). The between-subject variability necessitates dose individualization by considering patient-specific characteristics such as body weight, age, and ongoing organ maturation, which makes population pharmacokinetics a valuable approach.

Population pharmacokinetic analyses of vancomycin have been performed in a variety of disease conditions, including patients with sepsis or septic shock (Heffernan et al., 2019), critically ill patients (Llopis-Salvia and Jiménez-Torres, 2006; Udy et al., 2013; Escobar et al., 2014; Petejova et al., 2014), severely burnt patients (Yang et al., 2007; Dolton et al., 2010), cancer patients (Buelga et al., 2005; Omote et al., 2009; Al-Kofide et al., 2010), extremely obese patients (Grace, 2012; Adane et al., 2015), patients with various degrees of renal function (Rodvold et al., 1988; Staatz et al., 2006; Kim et al., 2019; Chu et al., 2020), patients with peritonitis (Montanes Pauls et al., 2011), and during hydrocephalic shunt prophylaxis (Leroux et al., 1990). Population pharmacokinetics have also been widely studied in different age groups, including geriatric patients (Usman et al., 2018; Zhou et al., 2019) and pediatric patients under various clinical conditions (Anderson et al., 2007; Machado et al., 2007; Lo et al., 2010; Badran et al., 2011; Giachetto et al., 2011; Stockmann et al., 2013a; Mahmoud et al., 2014; Zhao et al., 2014; Moffett et al., 2019a; Moffett et al., 2019b; Moffett et al., 2019c). Moreover, studies in different ethnic groups are also available regarding the population pharmacokinetics of vancomycin (Purwonugroho et al., 2012; Deng et al., 2013; Zhou et al., 2019). Simulations of different dosage regimens to maintain trough concentrations of vancomycin between 10 and 20 mg/l have been performed in different clinical cases such as critically ill patients (Kovacevic et al., 2020), pneumonia patients (Yamamoto et al., 2009), and patients with hematological malignancies (Buelga et al., 2005). The covariates considered for dosing simulations in different studies have included high-volume hemofiltration (Escobar et al., 2014) serum cystatin C (Chung et al., 2013), and creatinine clearance (Purwonugroho et al., 2012). However, to the best of our knowledge, based on a literature search, no population pharmacokinetic study of vancomycin has been reported in Pakistani patients, and this is probably the first study on the population pharmacokinetics of vancomycin in Pakistani patients using a non-linear mixed effect (NONMEM) modeling approach. Pakistan, with more than 200 million people (Pakistan Bureau of Statistics, 2017), exhibits diversity in its population with regard to lifestyle, eating habits, genetic polymorphism, etc. Since no population pharmacokinetic study of vancomycin in Pakistani patients has been reported so far, this study aimed to develop a population pharmacokinetic model of vancomycin in adult Pakistani patients by using NONMEM^®^ and to evaluate the influence of different covariates on pharmacokinetic parameters. Furthermore, the simulations of dosage regimens were performed using the population pharmacokinetic model, which was developed using data obtained from Pakistani patients rather than from the literature.

## Materials and methods

### Patient selection and sampling

A total of 176 blood samples were collected from 58 patients admitted to the surgical unit of Lahore General Hospital, Pakistan, between August and December 2018. The study was approved by the Institutional Review Committee (IRC) for Biomedical Research of the University of Veterinary and Animal Sciences, Lahore, Pakistan, vide notification no. 018/IRC/BMR. The sample collection was conducted in accordance with the Declaration of Helsinki for medical research involving human subjects (World Medical Association, 2001). Only patients who underwent a major surgical procedure and were above the age of 18 years were included in the study. Written informed consent was obtained from all patients or their close relatives. Vancomycin was administered *via* IV infusion for 0.5 h. The dose selection and duration of infusion were at the discretion of the attending medical practitioners. Blood samples were collected at different time intervals after administration of the first dose of vancomycin, and the samples were centrifuged at 5,000 rpm for 5 min to separate the plasma, which was stored at −20°C until analysis. Patient demographics, including age, weight, sex, type of disease, and serum creatinine (SCR) were recorded from patient files, and creatinine clearance (CRCL) was calculated using the Cockcroft and Gault equation (Cockcroft and Gault, 1976). The patients demographics and sampling record is provided in Table 1.

**TABLE 1:** Patients and sample data.

Patient demographic data	Median (range)
No. of patients	58
Male (%)	39 (73.7)
Female (%)	19 (26.3)
Age (years)	54 (25–86)
Weight (kg)	75 (53–129)
SeCr (mg/dL)	0.935 (0.4–4.7)
CRCL (mL/min)	101.15 (15.9–177.2)
Types of patients	—
Patients with grossly contaminated wound	13 (34.2%)
Gas gangrene patients	18 (47.4%)
Patients with severe peritonitis	7 (18.4%)
Sample data	—
No. of samples	176
Samples per patient (average)	3 (1–7)
Dose (mg)	500–1,000
Concentration (mg/L)	23.5 (1.9–52.6)

### Sample analysis

The concentrations of vancomycin in individual samples were quantified at the Quality Operation Laboratory of the University of Veterinary and Animal Sciences, Lahore, using an already developed and validated HPLC method (Usman and Hempel, 2016). Briefly, separation was carried out using a C18 column (125 mm × 4.6 mm, 5 µm) with UV detection at 205 nm. The mobile phase was a combination of buffer (50 mM ammonium dihydrogen phosphate) at pH 2.2 and acetonitrile (88:12 v/v). The method was linear over the concentration range of 0.25–60 mg/l. The lower limit of quantification was 0.25 mg/l with relative standard deviations (RSD) of 17.8% and 3.03% for inter-day and intra-day precision, respectively.

### Population pharmacokinetic modeling

The population pharmacokinetic model was developed using nonlinear mixed effect modeling (NONMEM version 7.4.4) software provided by Icon Clinical Research LLC, New York, NY, USA, along with the Perl-speaks-NONMEM (PsN) toolkit (Lindbom et al., 2005). The execution and management of the model as well as report generation were performed with the aid of Pirana (Certara, Princeton, NJ, USA) (Keizer et al., 2011). The actual process of modeling began with the development of one-compartment and two-compartment base models using the first-order conditional estimation method with interaction (FOCE-I) to obtain pharmacokinetic parameter estimates without any covariates. The allometric scaling function was used to evaluate the relationship between body weight and vancomycin clearance (CL) as well as volume of distribution (Vd), with a coefficient of 0.75 for CL and 1 for Vd. The exponential random-effect model was used to describe the between-subject variability (BSV) on pharmacokinetic (PK) parameters, while additive, proportional, and combined residual error models were tested to describe the residual error between the observed and predicted concentrations of vancomycin.

### Covariate analysis

The influence of different covariates (age, sex, WT, SeCR, and CRCL) on CL and Vd was determined by stepwise covariate modeling (SCM) in which the covariates were included in the model by forward inclusion and backward elimination processes. During the forward inclusion step, all the tested covariates were added to the base model in a stepwise manner, and the influence on the objective function value (OFV) of the model was observed. For a tested covariate, a drop of 3.84 points in OFV (*α* = 0.05) between the two nested models was considered significant for inclusion. The process was repeated until no significant covariate was available for inclusion in the full model. During the backward elimination step, the added covariates in the full model were eliminated one by one, and an increase in OFV was observed with a stricter criterion for level of significance (*α* = 0.01). A rise in OFV ≥6.63 points was considered significant for the retention of covariates in the final model.

### Model evaluation

A visual observation of the goodness-of-fit plots was performed to evaluate the predictive performance of the final model. The goodness-of-fit plots included the scatterplots of observed concentrations (DV) versus population-predicted concentrations (PRED), DV versus individual predicted concentrations (IPRED), conditional weighted residuals (CWRES) versus PRED, and CWRES versus time after dose. The robustness and stability of the final model were evaluated by bootstrap analysis with 1,000 datasets generated by repeated sampling from the final model with different combinations of subjects. The median values of bootstrap estimates along with 95% confidence intervals (based on the 2.5th and 97.5th percentiles) were compared with the respective parameter estimates of the final model.

A visual predictive check (VPC) was performed to evaluate the predictive performance of the final model. The observed concentrations along with the median and 5th and 95th percentiles were overlaid on the 90% prediction interval (5th and 95th percentiles) of simulated concentrations obtained from 500 virtual datasets of the final model.

### Dosing simulations

As vancomycin is primarily eliminated *via* the kidney through glomerular filtration, dosing simulations were performed based on the renal status of the patients. For this purpose, four virtual patients were simulated with CRCL levels of 20, 60, 100, and 140 ml/min. Initially, the same dose [1,000 mg every 12 h (q12h)] was used to simulate 1,000 plasma concentrations at a steady state for all four virtual patients by keeping the target trough concentration range (TTCR) between 10 and 20 mg/l. After visual observation of simulated concentrations, the tailored dose was used, and plasma concentrations were simulated for 1,000 patients at each level of CRCL.

## Results

### Patient demographics and exploratory data analysis

The plasma concentration data for 58 patients (39 male and 19 female) with 176 samples were used for the development of the base model. The median age and body weight of the patients were 54 years and 75 kg, ranging from 25 to 86 years and 53–129 kg, respectively. The median serum creatinine level was 0.935 mg/dl with a range of 0.4–4.7 mg/dl, while the median creatinine clearance calculated by the Cockcroft and Gault equation was 101.15 ml/min, ranging from 15.9 to 177.2 ml/min. The majority of patients received a 1,000-mg dose of vancomycin administered over a 0.5 h infusion.

### Population PK modeling

One-and two-compartment models were used for the data analysis. However, the two-compartment model was not able to predict inter-compartmental clearance (Q) or volume of the peripheral compartment (V_2_) in a stable manner. Moreover, the values of the fixed-effect and random-effect parameters were also not stable in the two-compartment models (results not shown). Therefore, the one-compartment model with first-order elimination (ADVAN1 TRANS2) was used for the development of the base model. The initial value of OFV for the base model was 729.9 while the OFV of the final model after the inclusion of significant covariates was 670.1, which is 59.8 points less than the base model. The BSV for vancomycin CL and Vd was described by an exponential random effect, while residual variability between the observed and predicted vancomycin concentrations was described by a proportional error model. The BSV for vancomycin CL and Vd were 11.3% and 22.8%, respectively, while the additive error was 3.07.

### Covariate analysis

Stepwise covariate modeling showed that CRCL and patient body weight were significant covariates for vancomycin CL and reduced OFV by 42.01 and 9.13 points, respectively, after inclusion in the base model. In the final model, the values for vancomycin CL and Vd were 2.45 L/h and 22.6 L, respectively. The BSV for vancomycin CL decreased from 26.6% to 9.14% after the inclusion of covariates. Eq. 1 describes the influence of CRCL and body weight on vancomycin CL in the final model.

$$CL_{j}=2.45\times \mathrm{CLCRCL}\times \mathrm{CLWT}\times e^{\eta 1}$$ (1)

where CL_j_ is the vancomycin clearance for the *j*th individual, η_1_ is the BSV for vancomycin CL, and 2.45 is the median value of vancomycin CL for this population.

$$\mathrm{CLCRCL}=\left(1+0.0046\times \left(\mathrm{CRC}L_{j}-101.15\right)\right)$$ (2)

$$\mathrm{CLWT}=\left(1-0.011\times \left(WT_{j}-75\right)\right)$$ (3)

where CRCL_j_ and WT_j_ are the CRCL and weight of the *j*th individual while 101.15 and 75 are the median values of CRCL and weight in the population, respectively.

**FIGURE 1:**
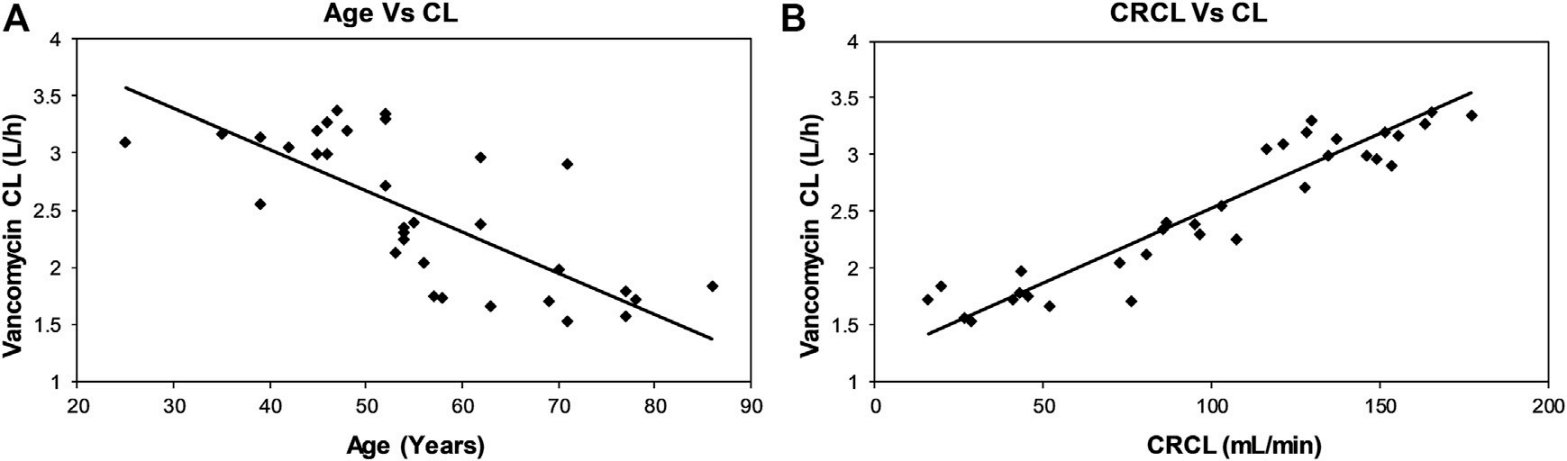
Scatterplots showing relationships between vancomycin CL and age **(A)** and CRCL **(B)** of the patients.

The inter-relationships between CL versus age and CL versus CRCL are shown in Figure 1. The CL of vancomycin decreased continuously with age (Figure 1A), while it increased in direct relation with CRCL (Figure 1B).

**FIGURE 2:**
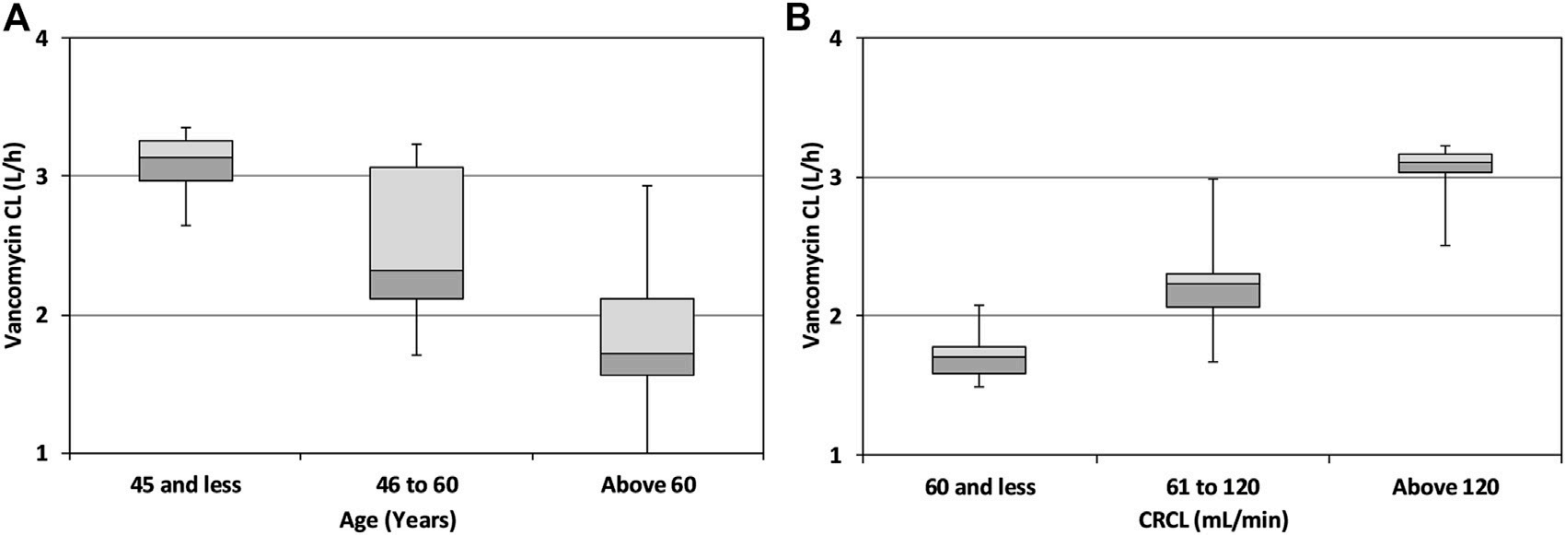
Box and whisker plots showing categorical change in vancomycin CL with age **(A)** and CRCL **(B)** of the patients after stratification into three groups.

Although the age of the patient was not a significant covariate for vancomycin CL, a categorical decline in vancomycin CL with increasing age was observed when age was stratified into three different groups: ≤45 years, 46–60 years, and ˃60 years (Figure 2A). A categorical increase in vancomycin CL was also observed with increasing CRCL when the patients were divided into three different groups based on CRCL: ≤60 ml/min, 60–120 ml/min, and ˃120 ml/min (Figure 2B).

### Model evaluation

**FIGURE 3:**
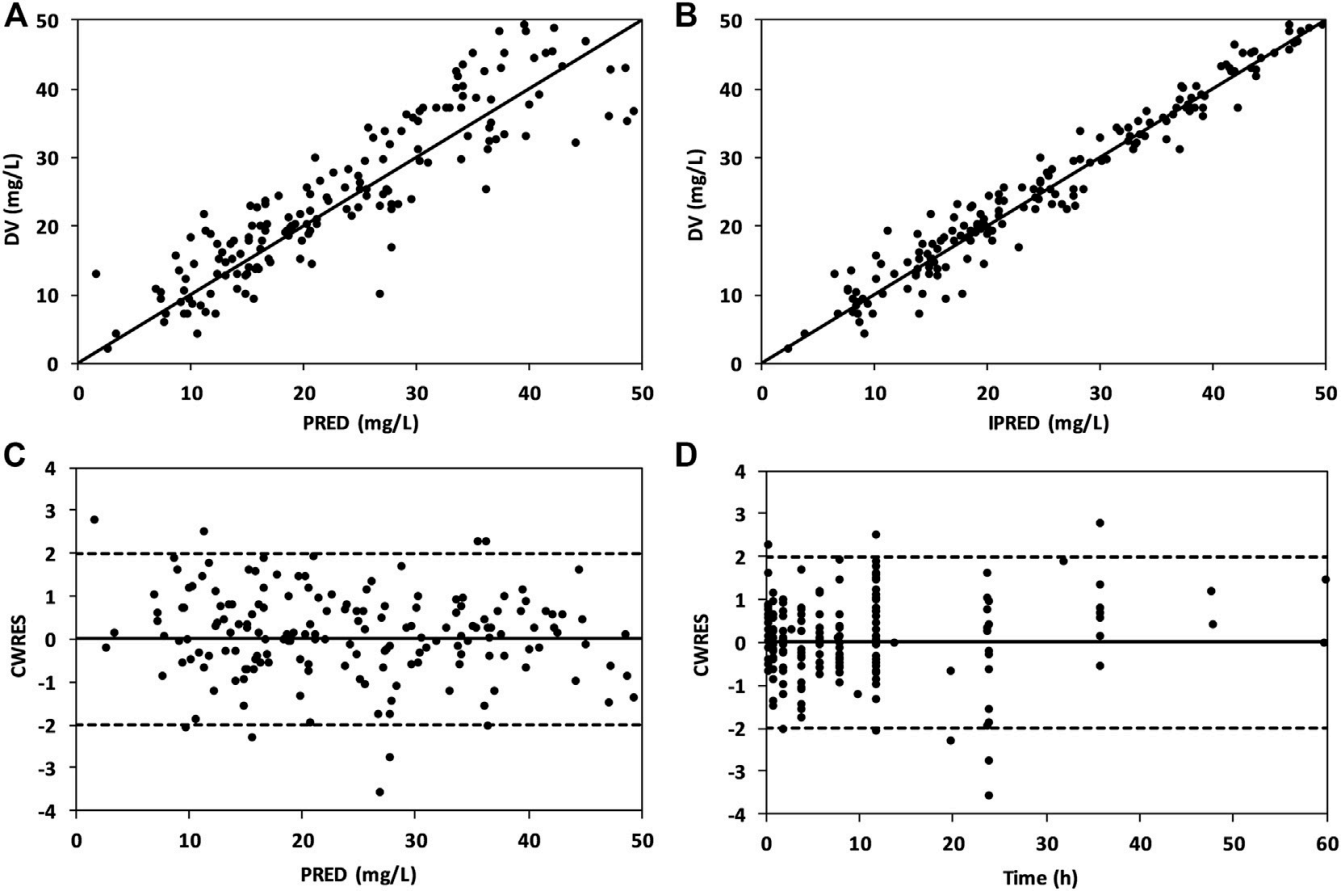
Goodness-of-fit plots of the final model showing DV vs. PRED **(A)**, DV vs. IPRED **(B)**, CWRES vs. PRED **(C)**, and CWRES vs. time after dose **(D)**.

The combined goodness-of-fit plots of DV versus PRED, DV versus IPRED, CWRES versus PRED, and CWRES versus time after dose are shown in Figures 3A–D. A uniform distribution of observed concentrations (DV) and predicted concentrations (PRED and IPRED) was observed around the line of identity (Figures 3A and B). Moreover, a random distribution of CWRES values around the zero line was observed, and 95% of the CWRES values were within the range of −2 to 2 in the final model (Figures 3C and D).

The parameter estimates of the final model compared with the median values of 1,000 bootstrap estimates along with 95% confidence intervals based on the 2.5th and 97.5th percentiles are given in Table 2. The values for bootstrap estimates were close to the parameter estimates of the final model, with a bias value of ≤7.25%.

**TABLE 2:** Population parameter estimates compared with 1,000 bootstrap estimates.

Parameter	Final estimate	RSE %	Bootstrap estimate	95% CI^a^	Bias %
OFV	670.1		659.9	540.7–757.5	1.51
CL (L/h)^b^	2.45	2	2.46	2.35–2.59	−0.21
V (L)	22.6	5	22.7	20.3–26.1	−0.39
CL-CRCL^c^	0.0046	13	0.0045	0.0033–0.0063	1.22
CL-WT^d^	0.011	10	0.0107	0.0087–0.013	2.15
BSV-CL (%)^e^	11.3	38	10.48	4.18–14.4	7.25
BSV-Vd^e^ (%)	22.8	51	21.8	13.7–53.1	4.38
Additive error	3.07	10	3.0	2.43–3.67	2.18

a 95% CI based on the 2.5th to 97.5th percentiles of distribution.

b Clearance at a CRCL of 101.15 (mL/min) and weight of 75 kg.

c Proportional change in CL with CRCL.

d Proportional change in CL with weight of the patients.

e Between subject variability of CL and Vd expressed in percentage coefficient of variation.

**FIGURE 4:**
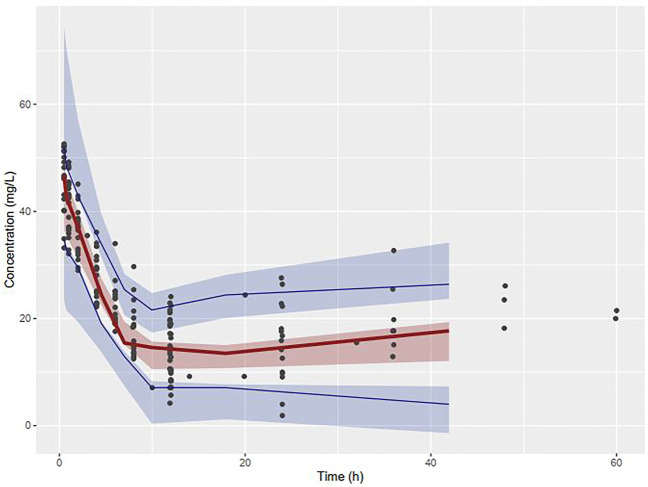
Visual Predictive Checks of observed concentrations of vancomycin placed over the median, 5th and 95th percentiles of simulated concentrations.

The VPC revealed that the observed concentrations of vancomycin along with median and 5th and 95th percentiles were within the 90% prediction interval of the simulated concentrations, as shown in Figure 4.

### Dosing simulations

**FIGURE 5:**
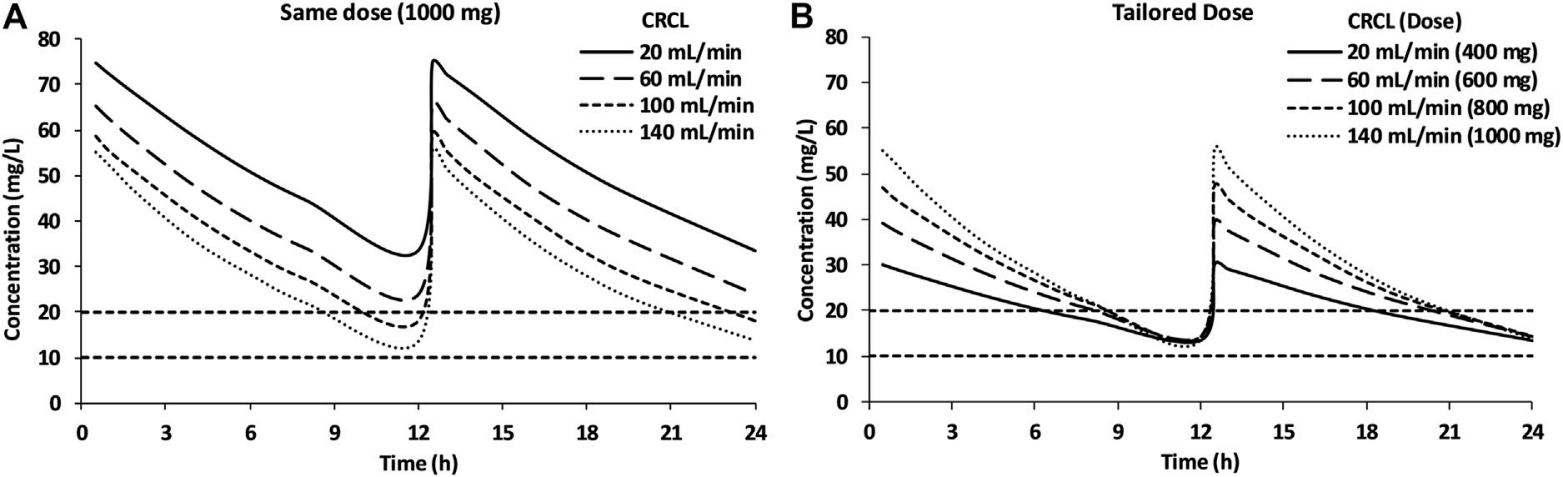
Simulations of the 1,000-mg q12h dose for patients with different renal status **(A)** and simulation of tailored dose based on the renal status of the patients **(B)**.

The results of the vancomycin dosing simulation at four levels of CRCL are shown in Figure 5. Administration of a similar dose (1,000 mg q12h) to all patients resulted in average vancomycin trough concentrations of 33.3, 23.9, 18.0, and 13.7 mg/l for patients with CRCL 20, 60, 100, and 140 ml/min, respectively (Table 3).

**TABLE 3:** Trough concentrations for common and tailored doses simulated for patients with different levels of creatinine clearance.

CRCL	Same dose (mg)	Simulated trough conc. (mg/L) Mean ± S.D	Tailored dose (mg)	Simulated trough conc. (mg/L) Mean ± S.D
20 ml/min	1,000	33.3 ± 7.54^a^	400	13.3 ± 3.01
60 ml/min	1,000	23.9 ± 5.73^a^	600	14.4 ± 3.44
100 ml/min	1,000	18.0 ± 4.56	800	14.4 ± 3.65
140 ml/min	1,000	13.7 ± 3.79	1,000	13.7 ± 3.79

a Above target trough concentration range (10–20 mg/l).

The vancomycin concentrations for patients with CRCL levels of 20 and 60 ml/min were above the TTCR, while patients with a CRCL of 100 ml/min maintained trough concentrations near the upper limit of TTCR (Figure 5A). After attempting multiple dosing regimens, 400, 600, 800, and 1,000 mg were found to be suitable for patients with CRCL of 20, 60, 100, and 140 ml/min, respectively, to maintain the trough concentration of vancomycin between 10 and 20 mg/l (Figure 5B).

## Discussion

Vancomycin is a narrow therapeutic index drug, and dose consideration is highly important for safe and effective treatment outcomes. The identification of patient characteristics and the magnitude of variability among individuals that influence the pharmacokinetics of a drug in a particular population can be helpful for dose individualization. As 80%–90% of administered vancomycin is eliminated through the kidney (Matzke et al., 1984; Rodvold et al., 1988; Ducharme et al., 1994), patients with augmented renal function (as in the case of increased cardiac output in the early stage of severe sepsis) are at high risk of sub-therapeutic plasma concentrations of vancomycin, which may result in therapeutic failure. On the other hand, patients with decreased renal function are at a risk of drug accumulation and toxicity (Taccone et al., 2011; Varghese et al., 2011). The selection of a dosage regimen is important for the safe and effective use of drugs in a target patient population or, more specifically, in an individual patient (Owen and Fiedler-Kelly, 2014). Population pharmacokinetic modeling is the study of variability among individuals with respect to drug concentrations after the administration of clinically relevant dosage regimens (Aarons, 1991), and it can be used to construct dosing strategies for individual patients (Ette and Williams, 2004).

Many studies have been conducted on the population pharmacokinetics of vancomycin in different countries and under different conditions. The majority of the studies were carried out in high-income countries, and few were conducted in upper-middle-income countries (UMICs) (Lo et al., 2010; Purwonugroho et al., 2012; Lin et al., 2016; Chen et al., 2018; Li et al., 2018), while only one study was available in a lower-middle-income country (Abdel Hadi et al., 2016). To the best of our knowledge, this is the first study to investigate the population pharmacokinetics of vancomycin in Pakistani patients.

The description of data by one compartment with first-order elimination is in line with the majority of reported population pharmacokinetic studies (Kimura et al., 2004; Buelga et al., 2005; Anderson et al., 2007; Lo et al., 2010; Tanaka et al., 2010; Stockmann et al., 2013a; Udy et al., 2013; Zhao et al., 2014; Adane et al., 2015; Usman et al., 2018; Moffett et al., 2019c; Heffernan et al., 2019; Zhou et al., 2019). However, two-compartment models were used to determine the population pharmacokinetics of vancomycin in some studies (Grimsley and Thomson, 1999; Thomson et al., 2009; Beumier et al., 2013; Escobar et al., 2014). The mean value of vancomycin CL in Pakistani patients was 2.45 L/h, which is in close agreement with the reported CL values of 1.08–2.99 L/h in other studies (Buelga et al., 2005; Llopis-Salvia and Jiménez-Torres, 2006; Staatz et al., 2006; Thomson et al., 2009; Tanaka et al., 2010; Udy et al., 2013; Escobar et al., 2014; Usman et al., 2018). However, obese patients observed in different studies exhibited high values of vancomycin CL, from 6.54 to 11.8 L/h (Blouin et al., 1982; Bauer et al., 1998; Adane et al., 2015). The BSV for vancomycin CL in Pakistani patients was 11.3%, which is less than the reported range from 16% (Staatz et al., 2006) to 40.6% (Seay et al., 1994). However, a lower value of BSV for CL of 4.5% has been reported in a study conducted on 1812 patients with end-stage renal disease (ESRD) (Goti et al., 2018). In the covariate analysis, CRCL and patient weight were significant covariates for vancomycin CL. As the major route of vancomycin elimination is the kidney, changes in CRCL can influence vancomycin CL. Body weight is another covariate that significantly affected vancomycin CL in Pakistani patients, possibly due to the diversity in body weight of patients used in this study (53–129 kg with a median of 75 kg). A few studies have also reported the influence of age on CL (Revilla et al., 2010; Chung et al., 2013). In neonates and children, body weight (Kimura et al., 2004; Stockmann et al., 2013b; Zhao et al., 2014; Guilhaumou et al., 2016; Li et al., 2018), gestational age (Grimsley and Thomson, 1999; Kimura et al., 2004; Lo et al., 2010), and postnatal age (PNA) (Anderson et al., 2007; Zhao et al., 2014; Stockmann et al., 2015; Chen et al., 2018) have been reported as significant covariates for vancomycin CL. The Vd for vancomycin was 22.6 l (BSV 16.1%), as compared with a reported range in Vd from 11.8 l (Escobar et al., 2014) to 101 l (Lin et al., 2016). However, no covariate was shown to be significant for Vd of vancomycin in our population.

The simulations of vancomycin dosing based on the renal status of patients indicated that 800–1,000 mg of vancomycin administered every 12 h is sufficient for maintaining the trough concentration of vancomycin between 10 and 20 mg/l in patients with CRCL of 100 and 140 ml/min, while administration of the same dose to patients with compromised renal status may lead to the accumulation of vancomycin; therefore, a dose of 400 and 600 mg is recommended if the CRCL of the patients is 20 and 60 ml/min, respectively. The dosing simulations for vancomycin in patients with estimated GFR reduction have also been previously performed in a similar study in which a 1,000-mg q12h dose of vancomycin was simulated in patients with a mild, moderate, or severe reduction in GFR (Kovacevic et al., 2020). A dose of 1,000 mg q12h was also simulated based on the serum cystatin C levels of the patients in another study (Chung et al., 2013). Furthermore, the optimal loading dose of vancomycin was estimated as 25 mg/kg/day with a maintenance dose reduced by 30% in non-dialysis patients, while in patients on dialysis, the optimum loading dose was shown to be 15 mg/kg/day with a maintenance dose reduced by 60% of the loading dose (Goti et al., 2018).

## Conclusion

A population pharmacokinetic model was developed, and the pharmacokinetic parameters of vancomycin in adult Pakistani patients were comparable to those in other populations. The CRCL and body weight of patients were shown to be significant covariates for vancomycin CL in Pakistani patients. The developed model was used to simulate dosage regimens based on the CRCL of patients. The tailored doses of vancomycin in Pakistani patients were 400, 600, 800, and 1,000 mg twice daily for patients with CRCL of 20, 60, 100, and 140 ml/min, respectively.

## Data Availability

The raw data supporting the conclusions of this article will be made available by the authors, without undue reservation.
