# Supplementary material for: Dose Tailoring of Vancomycin Through Population Pharmacokinetic Modeling Among Surgical Patients in Pakistan
Source: Front Pharmacol. 2021 Nov 11;12:721819. doi: 10.3389/fphar.2021.721819 (PMC8632000; doi:10.3389/fphar.2021.721819)
Supplement: Supplementary file 1 [file DataSheet1.PDF]

Control Stream

```
$PROBLEM    VANCO_PK

$INPUT      ID TIME DV AMT DUR CMT RATE MDV EVID WT SCR CRCL AGE SEX

$DATA       DATA_SRGP_15.csv IGNORE=#

$SUBROUTINES ADVAN1 TRANS2

$PK

;;; CLWT-DEFINITION START

CLWT = ( 1 - THETA(5)*(WT - 75))

;;; CLWT-DEFINITION END

;;; CLCRCL-DEFINITION START

CLCRCL = ( 1 + THETA(4)*(CRCL - 101.15))

;;; CLCRCL-DEFINITION END

;;; CL-RELATION START

CLCOV=CLCRCL*CLWT

;;; CL-RELATION END

D1=DUR

TVCL=THETA(1)*(WT/70)**0.75

TVCL = CLCOV*TVCL

TVV=THETA(2)*(WT/75)**1

CL=TVCL*EXP(ETA(1))

V=TVV*EXP(ETA(2))

S1=V

$ERROR

IPRED = F

W=THETA(3)

IRES=DV-IPRED

IWRES=IRES/W

Y=F+W*ERR(1) ; Proportional error
```

```
$THETA  (0,2.4561,5) ; CL

(0,22.5662) ; V

(0,3.2) ; Proportional error

$THETA  (0, 0.0044, 0.012) ; CLCRCL

$THETA  (-0.019,0.010239,0.045) ; CLWT

$OMEGA  0.0148299  ;     IIV CL

0.0231973  ;      IIV V

$SIGMA  1  FIX

$ESTIMATION MAXEVAL=9999 PRINT=5 METHOD=1 INTER MSFO=.MSF

$COVARIANCE

$TABLE ID TIME AMT RATE MDV DV PRED IPRED CWRES IWRES IRES EVID W

CL V ETA1 CRCL WT AGE ONEHEADER NOPRINT

FILE=Final.mod.mytab15
```
